# Whole and Sprouted Cereals as Nutritional Modulators of the Gut Microbiota and Intestinal Inflammation in IBD

**DOI:** 10.3390/nu18152447

**Published:** 2026-07-27

**Authors:** Valentina Álvarez-Arraño, Karen Toledo-Stuardo, Marjo J. E. Campmans-Kuijpers, Fabien Magne, Marcela A. Hermoso

**Affiliations:** 1 Department of Gastroenterology and Hepatology, University Medical Centre Groningen (UMCG), 9713 GZ Groningen, The Netherlands; v.p.alvarez.arrano@umcg.nl (V.Á.-A.); m.j.e.campmans-kuijpers@umcg.nl (M.J.E.C.-K.); 2 Laboratory of Innate Immunity, Program of Immunology, Institute of Biomedical Sciences, Faculty of Medicine, University of Chile, Santiago 8380453, Chile; karentoledostuardo@gmail.com; 3 Laboratory of Immunogastroenterology, Department of Medicine, Faculty of Medicine, University of Chile, Santiago 8380453, Chile; 4 Microbiology and Mycology Program, Institute of Biomedical Sciences, Faculty of Medicine, University of Chile, Santiago 8380453, Chile; fmagne@uchile.cl

**Keywords:** sprouted grains, whole grain, gut microbiota, inflammation, inflammatory bowel disease, diet

## Abstract

Ulcerative colitis (UC) and Crohn’s disease (CD), known as inflammatory bowel diseases (IBDs), result from a complex interaction of genetic, immunological, microbial and environmental factors. There is growing evidence suggesting that imbalances in the gut microbiota, or dysbiosis, play a causal role in IBD and are strongly influenced by aspects of the Western lifestyle. Diet is an important modulator of gut health, and whole cereals have attracted attention for their potential to positively shape gut microbiota and intestinal function. Germination further enhances the content of prebiotic substrates and bioactive compounds such as polyphenols, GABA and fiber, which may modulate inflammation, oxidative stress and immune responses. In vitro and clinical studies suggest that these compounds may reduce pro-inflammatory cytokines and improve symptoms in IBD, although robust evidence in patients is still lacking. Here, we provide an overview of whole and sprouted cereals and their effects on inflammation and gut microbiota, with particular focus on IBD. We also discuss the potential of sprouted grains as a complementary approach to diet alongside conventional therapy, highlighting their possible support to gut health to mitigate inflammation. Finally, we emphasize the need for further well-designed clinical studies to confirm their therapeutic potential and to better understand the mechanisms underlying their beneficial effects.

## 1. Introduction

Inflammatory bowel disease (IBD), which includes Crohn’s disease (CD) and ulcerative colitis (UC), is a group of chronic gastrointestinal disorders characterized by a dysregulated immune response and alternating periods of active inflammation (flares) and clinical remission [1]. Disease activity varies over time and influences symptom severity, intestinal inflammation, nutritional status, and therapeutic management [2]. Both conditions differ in several clinical aspects, including the location and extent of intestinal involvement, disease activity, nutritional status and therapeutic management. CD can affect any part of the gastrointestinal tract, most commonly the terminal ileum and colon, whereas UC is restricted to the colon and typically present as proctitis, left side colitis, or extensive colitis [3,4]. Although the exact etiology of IBD remains unknown, it is believed to be multifactorial, involving a complex interaction between genetic predisposition and environmental factors. In this context, environmental and lifestyle factors—particularly diet—such as Western diet characterized by low fiber content and high consumption of sugars and fats, are believed to play a significant role in the development of IBD [5]. In 2017, there were 6.8 million cases of IBD globally [6], and it is expected that by 2030, 1% of the world’s population will be living with IBD [7]. Among the proposed mechanisms involved in the pathophysiology of IBD, are dysbiosis of the intestinal microbiota (IM), increased intestinal permeability, and elevated oxidative stress. Together, these factors contribute to the sustained activation of the immune system and the development of chronic inflammation [8]. Traditionally, treatment of IBD has focused on the use of aminosalicylates, anti-inflammatory drugs, corticosteroids, biologic therapies and antibiotics [9]. However, approximately one third of patients do not respond adequately to anti-TNF therapies or lose responsiveness over time, highlighting the need for further complementary therapeutic strategies [10,11,12]. In this context, non-pharmacological interventions, particularly those related to nutrition, have gained great relevance. Diet not only plays a key role in symptom management, but also contributes to maintaining the disease in remission when used alongside standard medical therapy [13]. Nutritional management should be individualized according to disease activity, nutritional status, and the patient’s therapeutic regimen. For example, exclusive enteral nutrition may be indicated during active disease, whereas dietary patterns such as the mediterranean diet are generally recommended during remission [2]. Consequently, various dietary strategies have been investigated, such as, regimens with different fiber contents (high or low), nutrients, such as omega-3 fatty acids, probiotics, prebiotics and antioxidants; but these all have contradictory results [14,15,16]. Despite growing interest in nutritional interventions for the treatment of IBD, there is still a need to discover foods with positive effects on intestinal inflammation, epithelial barrier integrity, and IM. In this context, cereals are an essential part of our diet providing the largest percentage of calories for the world’s population, being rich in fiber, phenolic compounds and micronutrients [17]. Whole grains in particular have associated beneficial effects on intestinal health, including promoting microbial diversity, enhancing short-chain fatty acids (SCFAs) production, improving gut barrier function, and reducing intestinal inflammation [18,19]. Recently, germination—the process that gives rise to sprouted grains—has been proposed as a strategy to enhance the nutritional and functional properties of grains by increasing their bioactive compounds, such as antioxidants and fermentable fiber. However, its potential as complementary dietary strategy alongside standard medical therapy for IBD has not yet been widely explored in the scientific literature. This review aims to summarize current knowledge of the role of sprouted cereals in gut health and explore its potential application as a functional food in the context of IBD.

## 2. Methodology

For this narrative review, a comprehensive literature search was conducted in the Web of Science and PubMed databases. The review focused on studies investigating germinated cereals and their potential anti-inflammatory effects and/or impact on the intestinal microbiota, using intestinal epithelial cell models, murine models IBD, or studies involving IBD patients carried out between 1995 and 2025. For human studies, no restrictions regarding age or sex were applied. The search strategy included controlled vocabulary as well as free terms such as “whole grain”, “cereals”, “grain”, “sprouted cereal”, “germinated grain”, inflammatory bowel disease, ulcerative colitis, Crohn disease, inflammation, gut microbiota, and microbiome. Boolean operators (“AND”, “OR”) were used to capture all relevant studies. Additional articles were identified through manual screening of reference lists from relevant reviews and studies. A summary of the studies included in this review, including study design, experimental model, and main findings, is provided in Supplementary Table S1.

## 3. Factors Involved in the Pathophysiology of IBD: Microbiota, Epithelial Barrier, Oxidative Stress and Immunity

Although the cause of IBD has not yet been determined, possible pathophysiological mechanisms have been proposed. These include genetic factors, dysbiosis of the IM, increased intestinal permeability and oxidative stress. All of which promote the activation of the immune system and contribute to the maintenance of chronic inflammatory state in patients [20]. The epithelium serves as a dynamic, selectively permeable barrier that maintains separation between the gut lumen and the mucosal compartment. It plays a dual role by allowing the absorption of nutrients and water, while preventing the translocation of harmful microorganisms and toxins [21]. This barrier is mainly composed of a single layer of epithelial cells covered by a mucus layer and reinforced by intercellular junctional complexes—tight junctions (TJs), adherens junctions, and desmosomes—that tightly regulate paracellular permeability [21,22]. TJs are located at the apical region of the epithelial cell junctional complex and are key regulators of paracellular permeability. TJs are composed of transmembrane proteins such as occludin, claudins, and junctional adhesion molecules (JAMs), which are connected to cytoplasmic scaffolding proteins like zonula occludens (ZO-1, ZO-2, ZO-3). These proteins anchor the junctional complex to the actin cytoskeleton, ensuring mechanical stability and selective permeability. The proper assembly and function of TJs are essential to maintain epithelial polarity and prevent luminal antigens, bacteria, and toxins from penetrating the mucosa [21,23]. In addition, epithelial cells continuously interact with immune and microbial signals, making the epithelial barrier a key determinant of intestinal homeostasis. However, patients with IBD exhibit increased intestinal permeability and reduced expression of TJ proteins [24,25], conditions that disrupt epithelial integrity and modify the mucosal environment, favoring microbial imbalance.

On the other hand, the IM is composed of a vast ecosystem of approximately 10 to 100 trillion microorganisms, including fungi, viruses, protozoa and bacteria, the latter being the most abundant. Approximately 90% of the bacterial community is dominated by the phyla Firmicutes and Bacteroidetes, while other predominant phyla include *Proteobacteria*, *Actinobacteria*, *Verrucomicrobia*, and *Fusobacteria* [26]. Most bacteria coexist in a symbiotic relationship with their host and perform multiple essential physiological functions, These include the fermentation of dietary fiber, resulting in the production of SCFAs, mainly acetate, butyrate and propionate, and the synthesis of B vitamins and vitamin K. Additionally, IM has the capacity to modulate the immune system and contributes to immune tolerance, through IL-10-producing RORγt+ FoxP3+ Tregs [27], helping to prevent excessive immune responses to harmless antigens, such as food or components of the microbial community [28]. Furthermore, it promotes the maintenance of the intestinal barrier. For example, *Bacteroides thetaiotaomicron* expresses several fucosidases that release fucose from host glycans, which in turn stimulates goblet cells to increase mucin secretion, demonstrating the ability of the microbiota to regulate mucus production [29].

Nevertheless, patients with IBD exhibit dysbiosis in their IM, characterized by an imbalance in microbial populations, particularly an increase in pathobionts—microorganisms that are usually harmless but can trigger inflammation in a dysbiotic environment [30]. In this sense, Vich Vila et al. characterized the gut microbiota composition through metagenomic sequencing of stool samples from IBD patients. Here, they found a decrease in several butyrate-producing bacteria such as *Faecalibacterium Prausnitzii,* a beneficial bacteria with anti-inflammatory properties [31]. Previous studies showed increased Bacteroides and Enterobacteriaceae (*Escherichia*/*Shigella*) in CD and UC patients capable of disrupting epithelium and promoting inflammation [32]. Other studies have shown that IBD patients had reduced fecal SCFAs (acetate, butyrate, propionate), with lower levels in UC than CD [33]. This is relevant because SCFAs mediate interactions between the intestinal microbiota and the immune system, thereby influencing the function of epithelial and immune cells. Their absorption in epithelial cells occurs through the passive diffusion by monocarboxylate-transporter 1 (MCT1/SLC16A1) and the sodium-coupled monocarboxylate-transporter 1 (SMCT1/SLC5A8) [34] and also through activation of three different G protein-coupled receptors; GPR41 (free fatty acid receptor 3; FFAR3), GPR43 (free fatty acid receptor 2; FFAR2), and GPR109A (hydroxycarboxylic acid receptor 2; HCAR2) [35]. In this way, within the cell they can modulate the function of key enzymes and transcription factors, including histone acetyltransferases, deacetylases [36], and the hypoxia-inducible factor (HIF) [37]. The regulation of these factors is crucial for maintaining intestinal homeostasis. Histone acetyltransferases and deacetylases control chromatin accessibility and, consequently, the expression of genes involved in immune tolerance and epithelial integrity. SCFAs, particularly butyrate, inhibits histone deacetylases, thereby enhancing the transcription of anti-inflammatory and barrier-protective genes. In parallel, stabilization of HIF by microbial metabolites contributes to epithelial repair and mucus production under physiological hypoxia, further reinforcing the intestinal barrier and limiting inflammation [38]. In the context of IBD, gut dysbiosis, the consequent reduction in SCFAs production and down regulation of their transporters [39] promotes the growth of pathobionts and a microbial imbalance leading to increased release of immunostimulatory microbial components, such as lipopolysaccharide (LPS), flagellin, and peptidoglycan [40]. These components, known as pathogen-associated molecular patterns (PAMPs), are recognized by the innate immune system through Toll-like receptors (TLRs) and NOD-like receptors (NODs) expressed in epithelial and immune cells [41,42]. This recognition activates pro-inflammatory signaling pathways, such as NF-κB and MAPK, leading to the release of cytokines (TNF-α, IL-1β, IL-6) that sustain chronic inflammatory responses [43].

**Figure 1:**
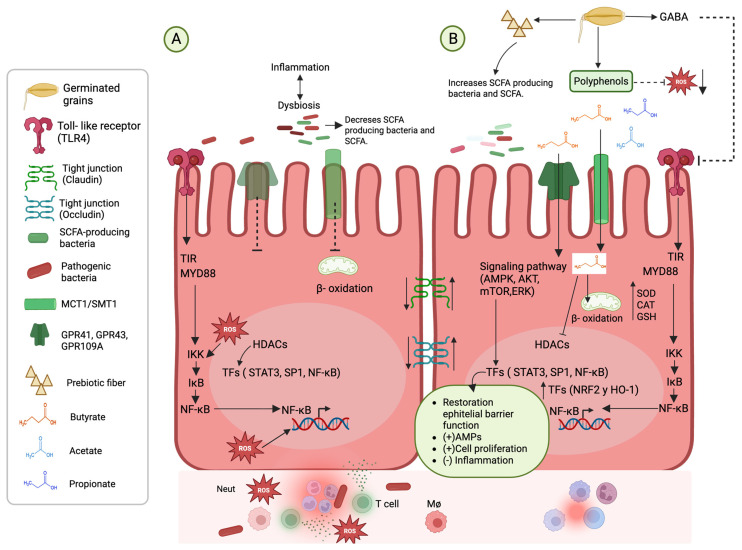
(**A**) Cellular and molecular mechanisms underlying intestinal inflammation in IBD. Microbial dysbiosis and oxidative stress contribute to the activation of intestinal immune responses. Reduced fermentation of dietary fiber, due to a loss of SCFAs-producing bacteria, leads to decreased luminal SCFA levels. Consequently, the expression of their receptors (GPRs and MCTs) is downregulated, resulting in limited HDAC inhibition. This favors SP1, NF-κB transcription factor activation and the subsequent production of pro-inflammatory cytokines. ROS promotes the activation of the IκB kinase (IKK) complex. Furthermore, ROS modulate the binding of NF-κB to DNA and enhance transcriptional activity. The loss of binding proteins allows bacteria to translocate from the apical zone to the lamina propria, which activates innate immune system cells and results in an inflammatory response. (**B**) Potential cellular and molecular mechanism of sprouted grains in the context of IBD. The prebiotic fiber contained in germinated grains can promote the growth of beneficial bacteria and increase the production of SCFAs. Butyrate can bind to its receptors and inhibit HDACs generating the same transcription factors promoting the restoring of the epithelial barrier. Moreover, butyrate can be oxidized by the mitochondria and enter the TCA cycle to produce energy. Polyphenols can reach the colon, where they can neutralize ROS and improve the endogenous antioxidant system (SOD, CAT, GSH) also being used as a substrate by IM. GABA inhibits the MyD88/TLR4 pathway, thereby increasing the expression of transcription factors that promote redox regulation. Taken together, this improves the functionality of the epithelial barrier, reducing inflammation. Created in https://BioRender.com. HDAcs: Histone Deacetylases; TFs: transcription factors; GABA: γ-aminobutyric acid; ROS: Reactive Oxygen Species; SCFAs: Short Chain Fatty Acids; Neut: Neutrophils; MΦ: Macrophages.

Another important mechanism contributing to mucosal damage in IBD is oxidative stress, which results from an imbalance between the production of radical species and the endogenous/exogenous antioxidant capacity [44]. During chronic inflammation, active immune cells —such as neutrophils and macrophages—and epithelial cells produce excessive amounts of reactive oxygen species (ROS) as part of the host defensive response. These ROS include superoxide (O_2_·^−^), hydroxyl radicals (HO·), peroxyl (RO_2_·), alkoxyl (RO·), hydroperoxyl (HO_2_·), and lipid hydroperoxides. In addition, reactive non-radical oxygen compounds like singlet oxygen (^1^O_2_) are generated [45]. These molecules are detected like a DAMP (damage associated molecular patterns) and contribute to the activation of TLRs (e.g., TLR4), favoring the activation of pro-inflammatory pathways, especially that of NF-κB, a key regulator sensitive to ROS. Its activation involves the release of NF-κB from its inhibitor IκB, allowing it to enter the nucleus and induce pro-inflammatory genes [46]. However, when these species are not adequately neutralized by the antioxidant system, damaged cellular components can be observed such as lipids, proteins, and DNA [47]. In addition, a decrease in the endogenous antioxidant enzymes Superoxide dismutase (SOD), Catalase (CAT) and Glutathione peroxidase (GPx) has been observed in patients with IBD (See Figure 1A for more details) [45]. This leads to epithelial damage, barrier disruption, and amplification of inflammation. In IBD patients, oxidative stress in the intestinal mucosa is observed as both a consequence and a driver of persistent inflammation [48].

## 4. Whole Grain Cereals

Cereals, which belong to the grass family (Poaceae), produce highly nutritious seeds or grains and are a fundamental part of the human diet and thus a staple food having significant health benefits. Cereals cover about 60% of the world’s agricultural land and are a major source of food energy, both for human consumption and animal feed [49]. According to the Whole Grains Council, whole grain foods are those that retain all the essential parts of the grain, bran, germ and endosperm, and maintain the nutrients in the original proportions present in the seed [50]. Whole grain flours are known to be more nutritious than refined flours because they retain the bran and germ of the grain, components that are removed during the refining process. They provide vitamins, minerals, antioxidants and fiber, with whole grain products rich in carbohydrates, protein, fiber, omega-3 fatty acids, vitamin B complex, phosphorus, magnesium, zinc and vitamins B complex and E [51]. They also contain bioactive compounds such as flavonoids, phenolic acids, carotenoids and lignans, the composition of which varies according to species, variety and growing conditions [52]. Moreover, whole grains contain natural polyphenols, such as flavonoids, phenolic acids and lignans, with anti-inflammatory, antioxidant and antibacterial properties that benefit gastrointestinal health. In addition, their consumption is associated with a reduced risk of chronic diseases such as cancer, type 2 diabetes, cardiovascular disease and obesity [52,53]. Some cereals have nutritional and sensory limitations due to protein of low biological quality, due to lysine (essential amino acid) deficiency, coarse texture that makes digestion difficult and reduced nutrient bioavailability due to the presence of anti-nutritional compounds [49]. These anti-nutrients include phytates, lectins, saponins, enzyme inhibitors, tannins, oxalates and phytoestrogens. Phytic acid can bind to minerals such as zinc, calcium and iron, decreasing their bioavailability [49]. However, despite their nutritional benefits, whole grains can present challenges in terms of digestibility and nutrient bioavailability due to the presence of anti-nutrients. Germination offers a promising solution to overcome these limitations and improve the nutritional quality of grains (described in detail below) [54]. Nevertheless, in the context of IBD, the consumption of gluten-containing cereals remains controversial, as some patients may experience Non-Celiac Gluten Sensitivity.

### 4.1. Non-Celiac Gluten Sensitivity (NCGS) and FODMAPs Diet

NCGS is a clinical disorder characterized by gastrointestinal and extraintestinal symptoms induced by wheat/or gluten-containing foods without the diagnoses of Celiac Disease and Wheat Allergy [55]. The prevalence in IBD patients is 25% being CD 24% and UC 27%, other studies have shown 27.6% [56]; however, a large American internet-based survey of 1647 IBD participants only found 4.9% [53]. Although current evidence is limited, studies suggest that the tolerance may depend on disease activity, how severe or structuring the disease is [56].

Also, 35% of patients with IBD exhibit gut symptoms even when the disease is in remission, and have low levels of inflammation [57], probably is related to a coexistent Irritable Bowel Syndrome (IBS) [58]. To diminish the symptoms associated with IBS some patients follow a diet low in fermentable oligosaccharides, disaccharides, monosaccharides, and polyols (FODMAPs) therefore reducing water and colonic gas and consequently, luminal distension [59]. However, IBS patients show decreased abundance of SCFAs producing bacteria, such as *Fecalibacterium prauznitzii* and bifidobacteria [60], associated with the production of anti-inflammatory cytokines such as IL-10 [61]. These considerations underscore the need for dietary approaches that alleviate gastrointestinal symptoms without compromising the intake of fermentable substrates essential for maintaining a beneficial gut microbiota.

### 4.2. Sprouted Grains

**Figure 2:**
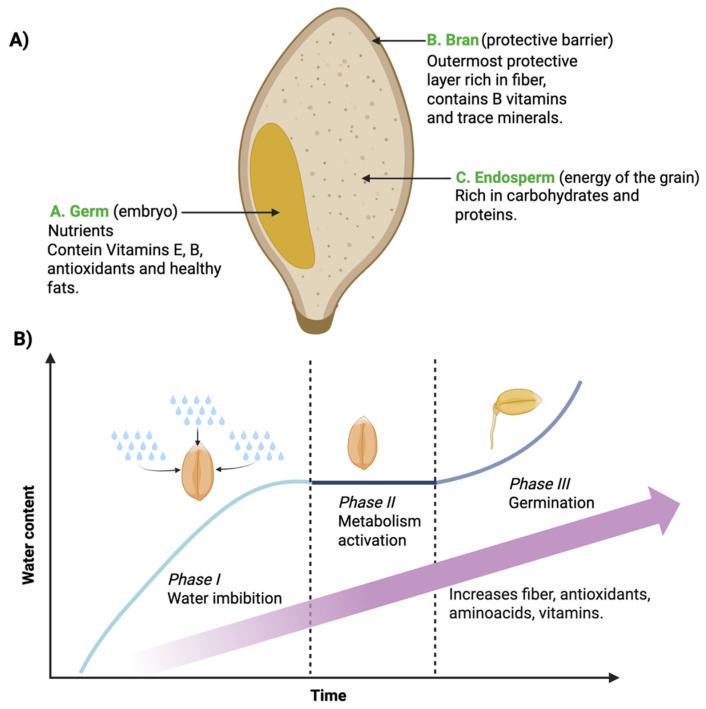
(**A**) Diagram of the grain is mainly composed of three structures: Bran, Germ and Endosperm, each of which has a metabolic and protective function. (**B**) Germination process, exemplified with a grain of wheat. This process has three fundamental stages, which are differentiated by the absorption of water from the grain, generating the metabolic changes necessary for the germination process. As time progresses, the content of polyphenols, amino acids, fiber, and vitamins increases. Created in https://BioRender.com.

Sprouted grains are products derived from the controlled germination of seeds, developed in water or other media, and harvested before leaf formation, which allows consumption in their entirety, including the seed. According to the definition established by The American Association of Cereal Chemists (AACC) and the United States Department of Agriculture (USDA), sprouted grains are “those malted or sprouted grains that retain the original bran, germ and endosperm intact” [62] (Figure 2A). These are classified as whole as long as the sprout does not exceed the length of the grain, and its nutritional values remain intact. In recent years, there has been a significant increase in the popularity of sprouted grains, driven by the growing interest in healthier, natural and minimally processed foods. These products can be consumed directly as fresh sprouts in salads or processed by drying and roasting to produce pastas, breads and beverages [63].

### 4.3. Germination Process

Germination is a biological process in which seeds restart their metabolism and begin to develop into new plants (Figure 2) [54]. The germination process can be divided into three critical stages; the first phase is rapid imbibition, in which the dry seeds absorb water at an accelerated rate until all their cellular structures and contents are fully hydrated. In the second phase, the rate of water absorption slows down and reaches a plateau, while the seed metabolism is significantly activated. The third phase is characterized by a further increase in water uptake, which allows cell elongation, culminating in the visible emergence of the sprout, marking the end of germination [64] (Figure 2B). This process induces changes in the macro- and micronutrient composition of the grain, which depend on factors such as germination time, humidity, temperature and grain genotype. In that sense, structural carbohydrates, particularly fiber, represent an essential component of whole grains. During germination, whole grains show significant increases in dietary fiber, antioxidants, inositol, phytic acid and free amino acids, especially γ-aminobutyric acid (GABA) [54,64]. In addition to fiber, germination enhances the antioxidant capacity of grains, with higher levels of phenolic compounds and related bioactives. For example, flavonoids increase by 2.25-fold in wheat. Polyphenols increase by 1.6-fold in barley [65,66]. Changes in the nutritional profile of sprouted grains are particularly relevant in IBD, where compounds such as antioxidants, fiber, GABA and reduction in anti-nutrients could help restore intestinal homeostasis and reduce inflammation.

## 5. Bioactive and Functional Components of Cereals and Sprouted Grains: Relevance in IBD

Diet has historically been recognized as an effective modulator of symptoms and progression of various diseases, and IBD is no exception. In fact, various dietary interventions support not only in symptom management, but also in inducing clinical remission in certain patients. Examples include exclusive enteral nutrition in children and adults with CD and the exclusion diet [2]. Additionally, because food is in direct contact with the gut, patients often experience gastrointestinal discomfort that leads them to restrict essential foods, ultimately resulting in nutrient deficiencies [67]. For these reasons, diet has become an important focus of research in the study of IBD. However, response to different dietary components can vary significantly between individuals, thus highlighting the need to personalize dietary recommendations rather than applying generalized approaches. In this context, sprouted grains have attracted growing interest because the process of sprouting increases the availability of various bioactive components, including soluble fiber, arabinoxylans, β-glucans, phenolic compounds, antioxidants, GABA, vitamins and free amino acids [54], that could have a positive influence on intestinal inflammation, epithelial function and microbiota. Understanding the role of these components is essential for evaluating their therapeutic potential in IBD.

### 5.1. Dietary Fiber and Prebiotic Effects

One of the key pillars proposed in the pathophysiology of IBD is intestinal dysbiosis [68]. In this regard, different dietary strategies have been explored with the aim of modulating and restoring their balance, with one of these strategies being prebiotics. According to the International Scientific Association for Probiotics and Prebiotics (ISAPP), prebiotics are defined as “a substrate that is selectively utilized by host microorganisms and confers a health benefit” [69]. This concept involves three fundamental elements: a specific substance, a physiologically beneficial effect, and a mechanism mediated by the microbiota. Although prebiotics are commonly associated with dietary fiber, not all fiber meet the criteria established by this definition. In addition, there are other compounds with prebiotic effects that do not come from fiber, such as certain polyphenols present in plant-based foods [69]. Prebiotics are found in whole grains, vegetables, fruits, and legumes. Among cereals, wheat, barley, rye, and oats stand out for their prebiotic substrate content, mainly fiber-derived compounds. For example, the wheat grain is composed of the germ, endosperm, and bran (Figure 2A). The bran contains prebiotic substances such as arabinoxylans (AX), while endosperm contains β-glucans. These compounds have been studied as a dietary strategy to improve gut health, thanks to their ability to modulate the composition and activity of the microbiota [70].

AXs are a non-starch polysaccharide of the grain cell wall with its backbone consisting of β-D-xylopyranose residues connected to α-L-furan arabinosyl substituents via C(O)-2, C(O)-3, or C(O)-2,3 bonds. Importantly, ferulic acid residues are esterified to the O-5 position of arabinose side chains, where they can undergo oxidative coupling to form diferulic bridges [71]. Recently, Huang et al. studied AX from barley bran in DSS-induced colitis in mice. Treatment with AX, alone or with 5-ASA, increased colonic SCFAs, enhanced intestinal barrier proteins, restored beneficial bacteria, and reduced pro-inflammatory cytokines. Similarly, wheat bran on human intestinal cell lines (Caco-2 and HT-29) showed an immunomodulatory effect characterized by reduced pro-inflammatory cytokines such as IL-8 and TNF-a [71]. These results support AX as a potential complementary dietary approach in UC [72]. The prebiotic effects of wheat flour were evaluated in vivo through bread consumption in patients with IBD in remission over 8 weeks. The results showed significant improvements in symptoms, with no significant changes in gut microbial diversity. No differences were observed in levels of inflammatory markers, such as C-reactive protein (CRP) or fecal calprotectin, suggesting that bread did not exacerbate inflammation [73]. However, fermentable arabinoxylans usually have a high degree of polymerization, consisting of long sugar chains. In addition, high-molecular-weight arabinoxylans have been linked to the restoration of beneficial bacteria. These structural characteristics appear to play a key role in determining their impact on gut health [74]. Moreover, the immunological properties of AX are strongly correlated with their structure, with a higher degree of arabinose substitution being potentially more effective in lowering inflammation in colon cancer cells [74].

β-Glucan, another prebiotic in cereals, is the main non-starch polysaccharide present in the cell walls of endosperm in cereal grains [75] (Figure 2A). Predominantly found in oats, barley, and wheat, structurally, they consist of a linear chain of β-(1→3) and β-(1→4) glycosidic linkages, compounds exhibiting a wide range of biological activities, including anti-inflammatory and immunomodulatory effects, among others [76]. For this reason, they have been studied in gastrointestinal disorders such as irritable bowel syndrome and have also been tested in IBD [77]. For example, in a mouse model of UC, the use of oat β-glucan reduced symptoms (weight loss, diarrhea, colon shortening) and decreased pro-inflammatory proteins such as TNF-α, IL-1β and IL-6, and the iNOS enzyme [78]. In a clinical trial with UC patients in remission, daily intake of oat bran significantly increased fecal butyrate levels and prevented deterioration of gastrointestinal symptoms [79].

Another important component present in cereals—especially in whole grains— is resistant starch, a type of carbohydrate considered prebiotic. It cannot be digested in the small intestine but can be fermented by the IM [80]. In animals, resistant starch increases SCFAs and reduces intestinal pH, hindering the growth of pathogens [81]. It has also been shown to reduce circulating cytokine levels and the clinical activity index in IBD [82,83]. The resistant starch content of cereal-based foods can be significantly modified by processing methods. Techniques such as cooking followed by cooling, or other forms of starch retrogradation increase the formation of structures resisting enzymatic digestion. This allows more fermentable substrate to reach the colon and thus promotes higher production of SCFAs [84,85]. Together, evidence suggest a potential biological rationale from cereal-derived prebiotics such as arabinoxylans, β-glucans, and resistant starch, on gut health through microbial fermentation and the subsequent production of SCFAs [75]. These metabolites contribute to the restoration of microbial balance, enhancement of intestinal barrier integrity, and modulation of immune responses [86]. Saccharolytic degradation of fiber in the gut depends on specific microbes rich in carbohydrate-active enzymes such as glycoside hydrolases and polysaccharide lyases [87]. Prebiotic fiber can be metabolized cooperatively through cross-feeding, where partially degraded polysaccharides by primary degraders serve as substrates for secondary fermenters. For instance, inulin-type fructans are extracellularly hydrolyzed by *Bifidobacteria* in the human colon, releasing sugars that can be utilized by butyrate-producing secondary degraders [86]. Although clinical studies remain limited, the available findings suggest their potential as complementary dietary strategies in IBD management. It is important to note that the physiological response to cereal-derived prebiotics is highly dependent on the initial composition of an individual’s gut microbiota [88].

### 5.2. Antioxidants and Modulation of Redox Balance

As previously mentioned, there are other prebiotic compounds not directly related to dietary fiber, such as antioxidants, whose main role is to prevent or delay oxidation of biological molecules [89]. Endogenous antioxidant enzymes such as: SOD, CAT and GPx provide the first defense against oxidative damage. Dietary antioxidants, including vitamins C and E, carotenoids, and flavonoids, act as a secondary defense [90]. Among them, ferulic acid (FA), a phenolic acid abundant in cereals, is notable for its strong antioxidant capacity. FA can activate the Keap1-Nrf2 signaling pathway, enhancing antioxidant activity and promoting the elimination of ROS [91]. During microbial fermentation, FA can be released by the action of ferulic acid esterases, increasing the host’s antioxidant capacity [92]. In parallel, fermentation also generates SCFAs, suggesting that FA and SCFAs may act synergistically to support intestinal health [93]. In fact, in a murine model treated with LPS, butyric acid and ferulic acid increased the concentration of IL-10 and *Bifidobacterium pseudocatenulatum* and suppressed the activation of the TLR4/NF-kB pathway [94]. Additionally, in LPS-treated Caco-2 cells, FA preserved tight junction gene expression, while in DSS-induced IBD mice it ameliorated weight loss, colon shortening, increased disease activity, and histological damage, indicating protective effects in IBD models [95]. Recent evidence has shown that FA significantly reduced IL-1β, IL-2, IL-6, and TNF-α levels, while enhancing occludin expression [96]. On the other hand, caffeic acid (CA), also present in cereals, has been reported to exert anti-inflammatory and antioxidant effects. In DSS-induced colitis mice, CA significantly reduced IL-6, TNF-α, IL-1β, IL-12, and ROS, while increasing IL-10 and occludin expression [97]. Cereal-derived compounds, particularly prebiotic fiber and phenolic acids may contribute to the maintenance of intestinal homeostasis by supporting microbial balance, epithelial integrity, and immune regulation. Although these bioactive substances show promising effects in IBD, their impact depends on factors such as molecular structure, fermentation dynamics, and nutrient bioavailability.

### 5.3. GABA

γ-aminobutyric is a non-protein amino acid naturally produced by plants, microbes, and humans [98]. It acts as the main inhibitory neurotransmitter in the central nervous system. Beyond the brain, GABA has been linked to immunomodulatory and anti-inflammatory effects [99]. Compared with controls, the levels of GABA were lower in serum and colon biopsies from patients with UC. This was also correlated with lower levels of GABA-producing bacteria in fecal samples from UC patients compared to the control group [100]. These findings suggest that GABA deficiency may contribute to hyperactivation of inflammatory pathways in UC. However, when GABA agonists have been tested in intestinal inflammation, they have been shown to reduce inflammation and oxidative stress. The mechanisms underlying its effect against oxidative stress suggested modulation of the TLR4/MyD88 pathway [101], supporting its potential role as a bioactive compound in sprouted grains.

### 5.4. Inositol Hexaphosphate (IP6)

Other bioactive component present in sprouted grains is Inositol hexaphosphate (IP6) also known as phytic acid, the main storage form of phosphorus in seeds, legumes, and grains [102]. In vitro studies suggest that IP6 may modulate immune responses by promoting M2a macrophage polarization. This shift is associated with increased expression of genes involved in anti-inflammatory processes and with reduced expression of pro-inflammatory cytokines (IL-1β, IL-6) in response to LPS stimulation [103]. IP6 also has preventative effects on metastasis of colorectal cancer in mice model [104]. Whereas evidence in patients with IBD remains limited. However, traditionally IP6 is considered an anti-nutrient due to its binding of minerals such as iron, zinc, calcium, and magnesium. During germination, phytase activity leads to the hydrolysis of IP6, reducing its anti-nutritional properties [105].

It is important to consider that the nutritional profile of grain depends on the species and germination conditions. While sprouted grains may provide a complex mixture of bioactive compounds with potential synergistic effects on inflammation and intestinal processes, current evidence is mainly derived from in vitro and mechanistic studies. In this context, the development of food enriched with sprouted grains, such as breads may offer opportunities for the development of functional foods. Nevertheless, additional clinical evidence is required to determine their impact on intestinal health and IBD outcomes.

## 6. Effect of Sprouted Grains on Gut Microbiota and Inflammation: Potential Role in IBD

Controlled clinical studies evaluating sprouted grains in patients with IBD remain scarce and are largely limited to older studies. Consequently, the current clinical evidence is insufficient to draw firm conclusions. Nevertheless, findings from in vitro and animal studies provide valuable mechanistic insights that help contextualize and support the interpretation of clinical observations.

Sprouted grains, due to their enhanced content of fermentable fiber and bioactive compounds, have been investigated for their potential role in modulating gut microbiota and intestinal inflammation, key components in the pathophysiology of IBD. One of the earliest studies was conducted by Kanauchi et al., who administered a diet containing germinated barley foodstuff (GBF) or cellulose (control) for 13 days in a murine model of IBD. GBF significantly increased butyrate-producing bacterial activity, reduced fecal bleeding, and downregulated pro-inflammatory markers such as IL-8 and NF-κB activity [106]. These findings suggest the importance of fiber fermentability, as GBF—unlike cellulose—serves as a substrate for microbial fermentation, leading to beneficial metabolic and immunological outcomes. In this context, studies evaluating the chemical composition and nutritional value of germinated barley have reported variable results regarding fiber content. While several analyses indicate an increase in crude fiber, other studies have shown a decrease or no significant change, likely due to differences in germination conditions, grain varieties, and analytical methods [107]. However, barley and oats are among the cereals with the highest natural concentrations of β-glucans, a type of soluble fiber with well-documented prebiotic effects [108]. Both in vitro and in vivo studies have demonstrated that it may modulate the composition of the gut microbiota and enhance the production of SCFAs, particularly butyrate, which plays a key role in maintaining intestinal homeostasis and reducing inflammation [109,110,111,112] (Figure 1B). In addition, germination has been shown to increase the antioxidant activity and flavonoid content of cereals, which may further contribute to their protective effects against intestinal oxidative stress and inflammation [113]. Subsequently, Kanauchi et al. [114] evaluated the dose-dependent effects of GBF (0–10%) in a DSS-induced colitis model in rats. Diets containing more than 6% GBF significantly elevated butyrate production in the large intestine. This increase in microbial fermentation was associated with remarkable protection of the intestinal mucosa and an almost complete prevention of hematochezia, underscoring the therapeutic potential of GBF in preserving epithelial barrier function during intestinal inflammation [114]. Butyrate exerts its anti-inflammatory effects through multiple mechanisms including the inhibition of histone deacetylases (HDACs), activation of G-protein coupled receptors (e.g., GPR43, GPR109A) (Figure 1B). Additionally, it enhances the expression of tight junction proteins and mucins, contributing to the maintenance of the intestinal epithelial barrier [37]. Faghfoori et al. provided clinical evidence by evaluating the effect of consuming 20 g/day of GBF for 8 weeks in patients with UC in remission, compared to baseline, GBF intake significantly reduced serum concentrations of IL-6 and IL-8 [115]. These findings support the anti-inflammatory potential of GBF in human IBD [114]. In addition, they tested the effect of GBF and its effect on serum CRP and clinical sign levels. After 2 months of intervention, this resulted in a significant decrease in CRP, abdominal pain and cramping in the GBF group [115]. Beyond fiber-mediated effects, the increased antioxidant capacity and flavonoid content of sprouted grains may also contribute to their anti-inflammatory potential. Flavonoids can scavenge ROS, modulate redox-sensitive transcription factors such as NF-κB, and suppress the production of pro-inflammatory cytokines, thereby attenuating intestinal inflammation [116]. Other interventions included the mixture of GBF plus *Clostridium butyricum* in mice with DSS-induced UC, which after 8 days of interventions prevented bloody diarrhea and mucosal damage [117]. These results show that the use of sprouted grains in combination with certain strains of bacteria can improve the damage caused by the disease. Sprouted wheat was also tested for anti-inflammatory effects in murine macrophage cell line RAW264.7. The strongest anti-inflammatory effect of wheat sprouts was observed after 4 days of germination, with marked reductions in TNF-α and IL-6, highlighting time as the key factor in cytokine inhibition [118].

Overall, the available in vitro animal and limited evidence in humans suggests that sprouted grains could have anti-inflammatory and barrier-protective effects through both microbiota-dependent and independent mechanisms. Their enhanced fermentable fiber content may promote SCFAs production—particularly butyrate—which supports epithelial integrity and immune regulation, while the increased levels of antioxidants and flavonoids further mitigate oxidative and inflammatory damage. Although most findings highlight germinated barley as a promising therapeutic adjunct in IBD, differences in germination conditions, grain type, and intervention design underscore the need for standardized approaches to fully elucidate the molecular pathways involved and optimize their clinical applicability. Additionally, for future trials in IBD patients it is necessary to mention the grain type, how many hours it was germinated, temperature and humidity. Additionally, it would be useful to mention study biomarkers, endoscopic findings, patient-reported outcomes, and microbiome endpoints to identify the optimal germination conditions that enhance the nutritional composition and bioactive profile of the grains, thereby maximizing their potential biological effects.

### Limitations, Uncertainties and Translational Considerations

The main limitations of this review is that much of the evidence discussed in this review originates from in vitro studies, animal models, or investigations using isolated bioactive compounds. While these findings provide valuable mechanistic insights, they do not necessarily reflect the effects of consuming whole sprouted cereal foods and should therefore be viewed as a basis for future research rather than direct support for dietary recommendations in IBD. Additionally, research on sprouted cereals is still in its early stages. The few human studies are relatively outdated, and recent high-quality clinical evidence is lacking. This introduces important uncertainties regarding their efficacy, safety, and consistency across different populations and disease states.

From a translational perspective, several barriers remain, including the lack of standardized germination and processing protocols, variability in the composition of bioactive compounds. In this context, differences between raw and processed sprouted grains may further influence their enzymatic activity and nutritional profile. In addition, microbiological safety during sprouting remains a relevant concern, because warm and humid conditions required for germination and many sources pre-and post- harvest may increase the risk of microbial contamination [64], especially *E. coli*, *L. monocytogenes*, and *salmonella* [119]. This issue is particularly relevant for patients with IBD receiving immunosuppressive therapies, as they are at higher risk of intestinal or systemic infections, including *Salmonella* spp. and *L. monocytogens* [120]. This highlights the need for strict safety and quality control before sprouted cereals can be considered in dietary strategies.

Moreover, potential inter-individual variability and disease activity may further influence patient responses, highlighting the need for caution when extrapolating in vitro findings to dietary recommendations. Patients with stenosing Crohn’s disease may require personalized dietary counseling, as tolerance to fiber-containing foods can vary according to the extent and severity of intestinal strictures. Therefore, while sprouted cereals may represent a promising dietary strategy, current evidence remains insufficient to support definitive conclusions, and further well-designed clinical studies are required to clarify their role in both IBD patients and healthy populations.

## 7. Conclusions

Sprouted cereals offer a promising dietary approach to support gut health and modulate inflammation in patients with IBD. Germination enhances the content of prebiotic substrates and bioactive compounds, which have been shown in in vitro and limited clinical studies to reduce pro-inflammatory cytokines and improve disease-related outcomes. Despite these encouraging findings, robust clinical evidence is still lacking, and the precise mechanisms through which sprouted grains exert their beneficial effects remain to be fully elucidated. Future well-designed clinical trials are warranted to validate their therapeutic potential and to determine optimal strategies for integrating sprouted cereals into conventional dietary and medical management of IBD.

## Data Availability

The original contributions presented in this study are included in the article/Supplementary Material. Further inquiries can be directed to the corresponding author.

## Supplementary Materials

The following supporting information can be downloaded at https://www.mdpi.com/article/10.3390/nu18152447/s1, Table S1. Summary of intervention studies evaluating the effects of sprouted grains in experimental models and patients with inflammatory bowel disease.
