# Supplementary material for: Whole and Sprouted Cereals as Nutritional Modulators of the Gut Microbiota and Intestinal Inflammation in IBD
Source: Nutrients. 2026 Jul 27;18(15):2447. doi: 10.3390/nu18152447 (PMC13468306; doi:10.3390/nu18152447)
Supplement: Supplementary file 1 [file nutrients-18-02447-s001.zip › nutrients-4361815-supplementary.pdf]

Supplementary Table S1. Summary of intervention studies evaluating the effects of sprouted grains in experimental models and patients with inflammatory bowel disease.

First Author, Year, Country	Study Population	Design	Intervention	Control	Type of Germinated Grain	Intervention Duration	Results
Kanauchi et al. (2002), Japan	18 patients with mildly to moderately active UC (n=11 GBF; n=7 control)	Multicenter, randomized, open-label control trial	20–30 g GBF daily + baseline anti-inflammatory therapy	Baseline anti-inflammatory therapy alone	Germinated Barley (aleurone and scutellum fractions)	4 weeks	Significant decrease in clinical activity index (scored via Lichtiger method) compared to control (P< 0.05). Endoscopic evaluation via Haring method supported clinical findings. Significant increase in fecal *Bifidobacterium* and *Eubacterium limosum*.
Kanauchi et al. (1999), Japan	5-week-old Sprague-Dawley rats (approx. 100g)	Experimental 3.5% DSS-induced colitis model	Diets with 4%–10% GBF; Exp 1 included 0.5% oral vs. 3% intracaecal sodium butyrate	Cellulose (CE) supplemented diet; 2 mL saline (intracaecal)	Germinated Barley (aleurone, scutellum, and germ fractions)	13–20 days	Dose-dependent reduction in mucosal damage (Cooper et al. crypt integrity score) and bloody diarrhea (significant at >6% GBF). Intracaecal, but not oral, butyrate reduced damage. Ibuprofen (beta-oxidation inhibitor) intensified damage and increased bacterial translocation to mesenteric lymph nodes (MLN).
Faghfoori et al. (2011) Irán	41 patients with UC in remission	Randomized pilot study (Primary Endpoint: Cytokines)	30 g GBF daily + standard drug therapy	Standard drug therapy alone	Germinated Barley (aleurone, scutellum, and germ)	2 months	Significant reduction in serum IL-6 (P = 0.034) and IL-8 (P=0.013). TNF-α showed a non-significant reduction (P= 0.18), while control group values demonstrated a notable rise (P= 0.08), highlighting divergent group responses.
Faghfoori et al. (2014) Irán	46 patients with UC in remission	Randomized, open-labeled pilot study (Primary Endpoint: CRP/Clinical Signs)	30 g GBF daily (divided into three 10g doses) + conventional medications	Conventional medications alone	Germinated Barley (aleurone, scutellum, and germ)	2 months	Significant reduction in serum C-reactive protein (CRP) (P= 0.017). Clinical symptoms of diarrhea and visible blood decreased but did not reach statistical significance; however, abdominal pain and cramping significantly improved (P= 0.016).
